# Elevated A2F bisect *N*-glycans of serum IgA reflect progression of liver fibrosis in patients with MASLD

**DOI:** 10.1007/s00535-024-02206-8

**Published:** 2025-01-24

**Authors:** Hisatoshi Hanamatsu, Goki Suda, Masatsugu Ohara, Koji Ogawa, Nobuharu Tamaki, Hayato Hikita, Hiroaki Haga, Shinya Maekawa, Masaya Sugiyama, Tatsuhiko Kakisaka, Masato Nakai, Takuya Sho, Nobuaki Miura, Masayuki Kurosaki, Yasuhiro Asahina, Akinobu Taketomi, Yoshiyuki Ueno, Tetsuo Takehara, Takashi Nishikaze, Jun-ichi Furukawa, Naoya Sakamoto

**Affiliations:** 1 Institute for Glyco-Core Research (iGCORE), Nagoya University, Aichi, Japan; 2 Department of Gastroenterology and Hepatology, Graduate School of Medicine, Hokkaido University, Hokkaido, Japan; 3 Department of Gastroenterology and Hepatology, Musashino Red Cross Hospital, Tokyo, Japan; 4 Department of Gastroenterology and Hepatology, Osaka University Graduate School of Medicine, Osaka, Japan; 5 Department of Gastroenterology, Faculty of Medicine, Yamagata University, Yamagata, Japan; 6 First Department of Internal Medicine, Faculty of Medicine, University of Yamanashi, Yamanashi, Japan; 7 Department of Viral Pathogenesis and Controls, National Center for Global Health and Medicine, Tokyo, Japan; 8 Department of Gastroenterological Surgery I, Graduate School of Medicine, Hokkaido University, Hokkaido, Japan; 9 Department of Gastroenterology and Hepatology, Tokyo Medical and Dental University, Tokyo, Japan; 10 Solutions COE, Analytical and Measuring Instruments Division, Shimadzu Corporation, Kyoto, Japan; 11 Department of Orthopaedic Surgery, Faculty of Medicine and Graduate School of Medicine, Hokkaido University, Hokkaido, Japan

**Keywords:** Metabolic dysfunction-associated steatotic liver disease (MASLD), Metabolic dysfunction-associated steatohepatitis (MASH), Glycomics, Mass spectrometry

## Abstract

**Background:**

Advanced liver fibrosis in cases of metabolic dysfunction-associated steatotic liver disease (MASLD) leads to cirrhosis and hepatocellular carcinoma. The current gold standard for liver fibrosis is invasive liver biopsy. Therefore, a less invasive biomarker that accurately reflects the stage of liver fibrosis is highly desirable.

**Methods:**

This study enrolled 269 patients with liver biopsy-proven MASLD. Patients were divided into three groups (F0/1 (*n* = 41/85), F2 (*n* = 47), and F3/4 (*n* = 72/24)) according to fibrosis stage. We performed serum *N*-glycomics and identified glycan biomarker for fibrosis stage. Moreover, we explored the carrier proteins and developed a sandwich ELISA to measure *N*-glycosylation changes of carrier protein.

**Results:**

Comprehensive *N*-glycomic analysis revealed significant changes in the expression of A2F bisect and its precursors as fibrosis progressed. The sum of neutral *N*-glycans carrying bisecting GlcNAc and core Fuc (neutral sum) had a better diagnostic performance to evaluate advanced liver fibrosis (AUC = 0.804) than conventional parameters (FIB4 index, aspartate aminotransferase-to-alanine aminotransferase ratio (AAR), and serum level of Mac-2-binding protein glycol isomer (M2BPGi). The combination of the neutral sum and FIB4 index enhanced diagnostic performance (AUC = 0.840). IgM, IgA, and complement C3 were identified as carrier proteins with A2F bisect *N*-glycan. A sandwich ELISA based on *N*-glycans carrying bisecting GlcNAc and IgA showed similar diagnostic performance than the neutral sum.

**Conclusions:**

A2F bisect *N*-glycan and its precursors are promising candidate biomarkers for advanced fibrosis in MASLD patients. Analysis of these glycan alterations on IgA may have the potential to serve as a novel ELISA diagnostic tool for MASLD in routine clinical practice.

**Clinical trial number:**

UMIN000030720.

**Supplementary Information:**

The online version contains supplementary material available at 10.1007/s00535-024-02206-8.

## Introduction

Metabolic dysfunction-associated steatotic liver disease (MASLD), a hepatic manifestation of obesity, diabetes mellitus, and dyslipidemia in the absence of significant alcohol consumption, is a prominent contributor to both liver-related morbidity and mortality, and has a significant impact on public health. The prevalence of MASLD is estimated to be around 30% worldwide [1–3]. MASLD is classified as either a progressive form (metabolic dysfunction-associated steatohepatitis (MASH)) or a non-progressive form (metabolic dysfunction-associated steatotic liver (MASL)). Since liver fibrosis is the most important prognostic factor for MASLD [3,4], accurate diagnosis of progression is crucial; however, the gold standard for accurately assessing liver fibrosis is liver biopsy, which is an invasive procedure that is both painful and associated with various complications [5]. Moreover, sampling error can lead to a false diagnosis, and histologic examination of the biopsy must be conducted by a specialized hepatologist to avoid intra- and inter-observer errors [6]. Although ultrasound, computed tomography, and magnetic resonance imaging are non-invasive diagnostic procedures, it is difficult to distinguish the progression of liver fibrosis in MASLD using imaging procedures alone [7,8]. Therefore, a less invasive and sensitive biomarker that reflects the progression of liver fibrosis is highly desirable.

The surface of mammalian cells is coated with a dense layer of glycocalyx comprising glycoproteins and glycolipids. Protein glycosylation, one of the most common post-translational modifications, plays an important role in many biological processes, including cell differentiation, cell adhesion, intermolecular interactions, and regulation of signaling pathways [9,10]. More than 50% of proteins in human serum/plasma are glycosylated [11]. Glycosylation can affect the biological activity of proteins, as well as their stability and transport to the cell surface; however [12], glycosylation patterns can alter markedly in response to various diseases such as autoimmune disorders, cancer, chronic inflammatory diseases, and viral infections [13]. Glycoproteins such as carbohydrate antigen 19-9 (CA19-9), CA125, prostate-specific antigen (PSA), and alpha-fetoprotein (AFP-L3) are used as cancer biomarkers in clinical practice, and detection of core-type fucosylated or multi-sialylated LacdiNAc structures on PSA has the potential to improve diagnostic or prognostic performance [14,15]. Therefore, we developed a glycoblotting method that allows rapid and quantitative glycome analysis, and found alterations in the expression of several *N*-glycans in the serum of patients with hepatocellular carcinoma [16]. Furthermore, total glycome analysis, which includes *N*-glycans, glycosphingolipids (GSLs), free oligosaccharides (fOS), and glycosaminoglycans (GAGs), identified novel glycan-related candidate biomarkers in various biological samples [16–19].

We also developed a method involving sialic acid linkage-specific alkylamidation (SALSA) of *N*- and GSL-glycans via lactone ring-opening aminolysis [20]. The SALSA method allows sialic acid linkage isomers to be distinguished by mass spectrometry analysis. Combining the aminolysis-SALSA method with isotope labeling revealed alterations in the ratio of α2,3-linked sialoglycans with or without fucose residues during the progression of fibrosis in patients with NAFLD [21]. In the present study, we used these advanced glycomic techniques to analyze serum samples from MASLD patients and demonstrated that expression of A2F bisect *N*-glycan (di-sialylated, biantennary, with core fucose and bisecting GlcNAc) and its precursors increases during fibrosis progression. We also identified specific carrier proteins of A2F bisect *N*-glycan, meaning a simple sandwich Enzyme-Linked Immuno Sorbent Assay (ELISA) system can be used to diagnose liver fibrosis progression.

## Methods

### Patients

This study enrolled 269 patients with liver biopsy conducted MASLD, diagnosed according to the criteria as follows; defined as the presence of hepatic steatosis in conjunction with one cardiometabolic risk factor and no other discernible cause [2]. The patients were recruited at Hokkaido University Hospital and six participating institutions. All patients underwent percutaneous liver needle biopsy to diagnose fatty liver disease between 2005 and 2020. We typically performed liver biopsies using an 18-gauge automated biopsy gun (Monopty needle; Bard Biopsy Systems, Tempe, AZ) and generally obtained 1.5–2.5 cm of liver tissue for diagnosis. All biopsy specimens were embedded in paraffin blocks in accordance with standard procedures and then stained with hematoxylin and eosin, Masson’s trichrome stain, and Gitter stain prior to evaluation by a hepatopathologist blinded to the clinical data. Samples were investigated and quantified based on the NAFLD activity score (NAS) [22] for steatosis (0–3), lobular inflammation (0–3), and hepatocyte ballooning (0–2). Each fibrosis parameter was scored according to the fibrosis stage of the Brunt classification [23]: advanced fibrosis was defined as Brunt stage F3/4. Serum was collected within 3 days of liver biopsy and stored at − 80 °C until analysis. The exclusion criteria were as follows: daily alcohol consumption > 30 g for men or > 20 g for women, and the presence of another hepatic disease such as hepatitis B, hepatitis C, hepatocellular carcinoma, autoimmune hepatitis, primary biliary cholangitis, primary sclerosing cholangitis, hemochromatosis, Wilson's disease, or congestive liver disease. The study protocol complied with the ethical guidelines of the Declaration of Helsinki and was approved by the Institutional Review Board of Hokkaido University Hospital and each participating hospital. Written informed consent to participate in this study was obtained from each patient. This study is registered in the UMIN Clinical Trials Registry as UMIN000030720. The clinical characteristics of the MASLD patients are summarized in Table 1, with further details provided in the supplementary information.

**Table 1:** Clinical characteristics of the patients with MASLD

	F0/1 (*n* = 126)	F2 (*n* = 47)	F3/4 (*n* = 96)	*P* value
				F0/1/2 vs. F3/4
Male (%)	49.2%	27.7%	36.5%	0.302
Age (Yr)	48 ± 14	60 ± 9	62 ± 8	< 0.001
BMI (kg/m^2^)	30.2 ± 5.0	27.9 ± 4.2	29.5 ± 3.9	0.912
Plt (10^4^/μL)	23.0 ± 4.9	21.0 ± 5.1	16.5 ± 4.9	< 0.001
Albumin (g/dL)	4.3 ± 0.3	4.2 ± 0.3	4.0 ± 0.3	< 0.001
T-bil (mg/dL)	0.8 ± 0.3	0.7 ± 0.2	0.9 ± 0.4	0.137
AST (IU/L)	58 ± 28	70 ± 31	60 ± 21	0.272
ALT (IU/L)	96 ± 58	80 ± 37	62 ± 28	< 0.001
ChE (U/L)	372 ± 66	345 ± 66	299 ± 67	< 0.001
γ-GTP (IU/L)	93 ± 62	89 ± 56	83 ± 44	0.548
AFP (ng/mL)	3.8 ± 1.5	4.9 ± 2.1	5.7 ± 2.8	< 0.001
TG (mg/dL)	180 ± 84	149 ± 58	145 ± 55	0.151
LDL-C (mg/dL)	119 ± 28	108 ± 28	104 ± 24	0.018
HDL-C (mg/dL)	52 ± 12	54 ± 12	50 ± 10	0.491
CRP (mg/dL)	0.31 ± 0.26	0.20 ± 0.15	0.28 ± 0.22	0.626
HbA1c (%)	6.4 ± 0.9	6.4 ± 0.7	6.6 ± 0.8	0.011
FBS (mg/dL)	121 ± 28	116 ± 22	130 ± 29	0.007
IRI (μIU/L)	20.1 ± 13.2	21.9 ± 17.4	32.4 ± 31.0	0.250
HOMA-IR	6.5 ± 5.0	7.5 ± 7.1	12.3 ± 13.7	0.116
FIB4 index	1.54 ± 0.87	2.52 ± 1.19	3.47 ± 1.49	< 0.001
AAR	0.75 ± 0.25	0.94 ± 0.27	1.08 ± 0.25	< 0.001
M2BPGi (COI)	0.89 ± 0.47	1.31 ± 0.71	1.97 ± 1.15	< 0.001
Pathological findings				
Fibrosis (0/1/2/3/4)	41/85/0/0/0	0/0/47/0/0	0/0/0/72/24	

Clinical data, including sex, age, height, and weight, were obtained for each patient at the time of liver biopsy, and body mass index (BMI) was calculated as weight divided by height in meters squared. The following biochemical variables in serum were measured by a conventional automated analyzer: platelet count (Plt), albumin, total bilirubin, aspartate aminotransferase (AST), alanine aminotransferase (ALT), γ-glutamyltransferase (γGTP), cholinesterase (ChE), α-fetoprotein (AFP), triglycerides (TG), low-density lipoprotein LDL-cholesterol (LDL-C), high-density lipoprotein cholesterol (HDL-C), hemoglobin A1c (HbA1c), ferritin, C-reactive protein (CRP), fasting blood sugar (FBS), and immunoreactive insulin. Insulin resistance was evaluated based on the homeostasis model assessment and expressed as an index of the insulin resistance (HOMA-IR) value, calculated using the following equation: HOMA-IR value = fasting insulin (µU/mL) × fasting glucose (mg/dL)/405. The formula used to predict liver fibrosis from data obtained non-invasively was as reported previously: fibrosis 4 (FIB4) index = age × AST (IU/L) × Plt (× 10^9^/L)^−1^ × √ALT (IU/L)^−1^ [24]. The aspartate aminotransferase (AST)-to-alanine aminotransferase (ALT) ratio (AAR) was calculated as AST (IU/L)/ALT (IU/L) [25]. The serum level of Mac-2-binding protein glycol isomer (M2BPGi) was measured as a marker of liver fibrosis.

### Precipitation of glycoproteins from human serum

Glycoproteins were prepared by ethanol precipitation as described previously [17,26]. A detailed description is provided in the supplementary methods.

### Preparation of *N*-glycans by glycoblotting combined with aminolysis-SALSA

Preparation of serum *N*-glycans based on glycoblotting and aminolysis-SALSA was performed using the SweetBlot high-throughput, semi-automated work system (System Instruments Co., Tokyo, Japan) [16]. A detailed description is provided in the supplementary methods.

### Sandwich ELISA for detection of immunoglobulin A bearing neutral bisect *N-*glycans

By peptide mass fingerprinting (PMF) analysis, immunoglobulin A (IgA) was identified as one of the carrier proteins with A2F bisect *N*-glycans as shown in Supplementary Table S4. From the results, we constructed a sandwich ELISA system using an anti-human IgA antibody and PHA-E lectin. Briefly, ELISA plates (MaxiSorp Plate; Thermo Fisher Scientific, Japan) were coated with a mouse anti-human IgA antibody (0.4 μg/mL; Nordic-MUBio, Susteren, Netherlands). Next, 100 μL of each serum sample (diluted 1:8000 in PBS, pH 7.2/0.05% Tween 20) or standard IgA (0–350 ng/mL in PBS, pH 7.2/0.05% Tween 20) were added to the plate for 1 h at 25 °C. The wells were washed three times with PBS, pH 7.2/0.05% Tween 20, and then incubated with 100 μL of PHA-E-HRP for 1 h at 25 °C, followed by washing as described above. For color development, TMB was added for 30 min at 25 °C. After terminating the reaction with sulfuric acid, absorbance at 450 nm (OD450) was measured in a microplate reader. For correction, the OD630 value (reference absorbance at 630 nm) was subtracted from the OD450. The amount of the IgA carrying neutral bisect *N*-glycans was calculated from a calibration curve generated using human IgA.

### Statistics analysis

Continuous variables were analyzed using the Mann–Whitney *U* test, and categorical variables were analyzed using Fisher’s exact test. Multivariate logistic regression analysis with stepwise forward selection was performed using variables identified as significant (*P* < 0.05) in univariate analyses.

The diagnostic performance of the markers was assessed by analyzing receiver operating characteristic (ROC) curves. The probability of true positives (sensitivity) and true negatives (specificity), as well as the positive-predictive value (PPV) and negative-predictive value (NPV), were determined for the selected cut-off values, and the area under the ROC (AUC) was calculated for each index. Cut-off points were determined based on the optimum sum of the sensitivity and specificity. Statistical analyses were performed using GraphPad Prism version 8.4.3 (GraphPad Software, MA, USA), SPSS Statistics 24.0 (IBM Corp., Armonk, NY, USA), and EZR (Saitama Medical Center, Jichi Medical University, Saitama, Japan). A *p*-value of 0.05 was deemed significant.

## Results

### Characteristics of the MASLD patients

In total, 269 MASLD patients were enrolled in the study. Patients were divided into three groups, F0/1 (*n* = 41/85), F2 (*n* = 47), and F3/4 (*n* = 72/24) based on the pathological severity of fibrosis in liver biopsy specimens. As shown in Table 1, the F2 group had a lower proportion of males and a lower BMI than the F0/1 group. Age, AST, and AFP levels were significantly higher in the F2 and F3/4 groups than in the F0/1 group. FBS levels were significantly higher in the F3/4 groups than in the F0/1 and F2 groups. HbA1c levels were significantly higher in the F3/4 group than in the F0/1 group. Platelet counts fell significantly as liver fibrosis progressed. Albumin, ALT, and ChE in the F3/4 groups were significantly lower than in the F0/1 and F2 groups. TG and LDL-C levels in the F3/4 group were significantly lower than in the F0/1 group. There were no significant differences in T-Bil, γ-GTP, HDL-C, CRP, IRI, and HOMA-IR values among the five groups. All fibrosis prediction formulas (FIB4 index and AAR) and fibrosis markers (M2BPGi) were significantly higher in patients with progression of liver fibrosis.

### Comprehensive *N*-glycome analysis in the serum of patients with MASLD by glycoblotting combined with aminolysis-SALSA

**Fig. 1:**
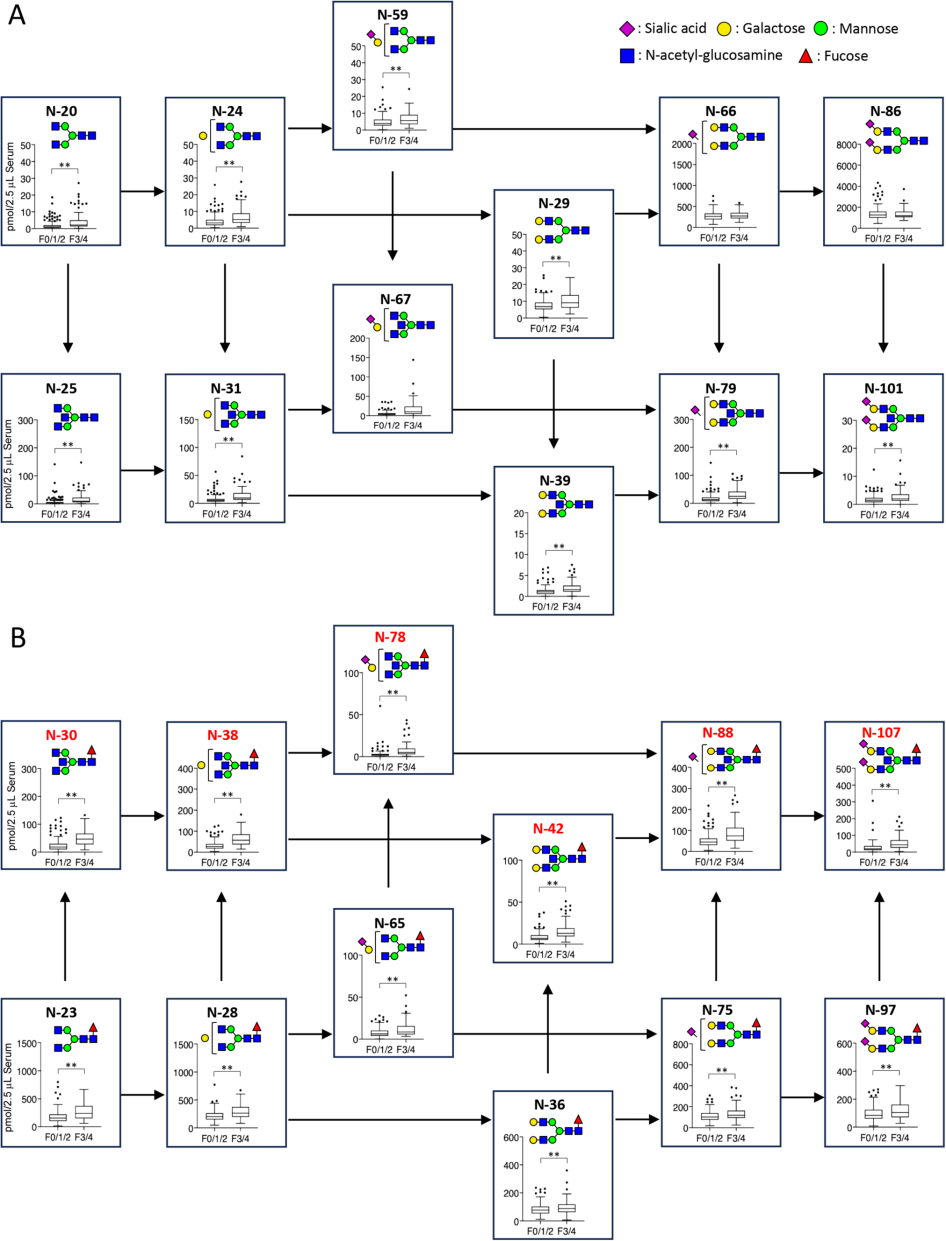
Possible biosynthetic pathway that generates A2F bisect *N*-glycan. The putative structures and expression levels of A2F bisect and its precursors are indicated. A Possible biosynthetic pathway of bisect N-glycan and its precursors. B Possible biosynthetic pathway of fucosylated bisect N-glycan and its precursors. Red characters denote A2F bisect, and precursors with bisecting GlcNAc and core fucose. Results are expressed as the mean ± S.D. *, 0.01 < *p* < 0.05; **, *p* < 0.01

Patients were divided into three groups, F0/1 (*n* = 126), F2 (*n *= 47), and F3/4 (*n* = 96), to explore the relationship between alterations in glycan expression and progression of fibrosis. After *N*-glycomic analysis, 138 types of *N*-glycan were observed in patient serum samples. The amount of each glycan according to the stage of fibrosis progression and the *p*-values are summarized in Supplementary Table S1. Whereas expression of total *N*-glycans and A2 glycan (N-86; (Hex)_2_(HexNAc)_2_(α2,6NeuAc)_2_ + (Man)_3_(GlcNAc)_2_), which is the most abundant form in serum, did not change, that of many individual *N-*glycans changed significantly as fibrosis progressed. When ranked in decreasing order of *p*-value derived from comparative analyses of the F0/1/2 and F3/4 groups (Supplementary Tables S2), A2F bisect (N-107; (Hex)_2_(HexNAc)_3_(Fuc)_1_(α2,6NeuAc)_2_ + (Man)_3_(GlcNAc)_2_) and its precursors occupied the top positions. Therefore, we first analyzed the biosynthetic pathway of A2F bisect glycan, including its expression levels (Fig. 1). In addition, we carried out a ROC analysis to evaluate the diagnostic utility of A2F bisect glycan and its precursor glycans for discriminating advanced liver fibrosis (F3/4) (Table 2). As shown in Fig. 1, expression of A2F bisect (N-107), A1F bisect (N-88), and monosialylated G1F bisect (N-78) glycans increased significantly in cases of advanced fibrosis, with AUC values of 0.754, 0.746, and 0.79, respectively. The expression levels of A2 bisect (N-101), A1 bisect (N-79), and monosialylated G1 bisect (N-67) glycans lacking core fucose tended to be higher in cases of advanced fibrosis; however, the AUC values were lower than those of fucosylated glycans. Furthermore, levels of A2 (N-86) and A1 (N-66) glycans lacking bisecting GlcNAc and core fucose did not change as fibrosis progressed (Supplementary Table S3).

**Table 2:** Diagnostic performance of A2F bisect, its precursors, and conventional markers for discrimination of advanced liver fibrosis

		*p* value	AUC	Cut-off	Sensitivity (%)	Specificity (%)	PPV (%)	NPV (%)
No	Class	F0/1/2 vs F3/4						
A2F bisect and precursors with bisecting GlcNAc and core fucose								
N-30	NBF	3.4E-14	0.792	28.4	75.0	74.0	15.8	38.5
N-38	NBF	3.7E-17	0.803	40.8	70.8	77.5	17.3	36.4
N-42	NBF	7.3E-13	0.764	9.7	72.9	70.5	17.6	42.1
N-78	ABF	2.2E-08	0.790	2.6	77.1	68.2	15.7	42.6
N-88	ABF	6.8E-11	0.746	64.7	66.7	78.6	19.0	36.6
N-107 (A2F bisect)	ABF	3.1E-09	0.754	21.2	85.4	53.8	13.1	49.4
Neutral sum (N-30, −38, −42)	NBF	3.9E-17	0.804	72.7	79.2	69.9	14.2	40.6
Acidic sum (N-78, −88, −107)	ABF	4.0E-11	0.762	93.3	71.9	71.7	17.9	41.5
Total sum (N-30, −38, −42, −78, −88, −107)	NABF	5.9E-15	0.795	173.8	75.0	72.8	16.0	39.5
Conventional markers								
FIB4 index		7.4E-13	0.799	1.8	85.4	64.7	11.1	42.7
AAR		2.9E-09	0.750	0.8	88.5	54.9	10.4	47.9
M2BPGi		4.3E-08	0.774	0.9	79.1	62.6	15.4	46.5

*N* Neutral, *A* Acidic, *B* Bisect, *F* Fucose

Regarding neutral *N*-glycans, expression of G2F bisect (N-42), G1F bisect (N-38), and G0F bisect (N-30) glycans containing bisecting GlcNAc and core fucose increased significantly as fibrosis progressed, with AUC values of 0.764, 0.803, and 0.792 respectively. Expression of G2 bisect (N-39), G1 bisect (N-31), and G0 bisect (N-25) glycans lacking core fucose also increased significantly as fibrosis progressed, but their AUC values were lower than those of fucosylated glycans. The AUC values of G2 (N-29), G1 (N-24), and G0 (N-20) glycans lacking bisecting GlcNAc and core fucose were lower than those of glycans containing bisecting GlcNAc (Supplementary Table S3).

Next, we categorized glycans into three groups to further evaluate the progression of fibrosis. Each of the three groups included both core fucose and bisecting GlcNAc residues as follows: (1) neutral *N*-glycans (Neutral sum; N-30, N-38, and N-42); (2) sialylated *N*-glycans (Acidic sum; N-78, N-88, and N-107); and (3) the total amount of *N*-glycans (Total sum). The expression levels of all three groups increased significantly as fibrosis progressed (Supplementary Figure S1); the AUC values for the neutral sum, acidic sum, and total sum groups were 0.804, 0.762, and 0.795, respectively (Table 2).

### Correlation between expression of glycans carrying bisecting GlcNAc and core fucose and conventional parameters of liver fibrosis

**Fig. 2:**
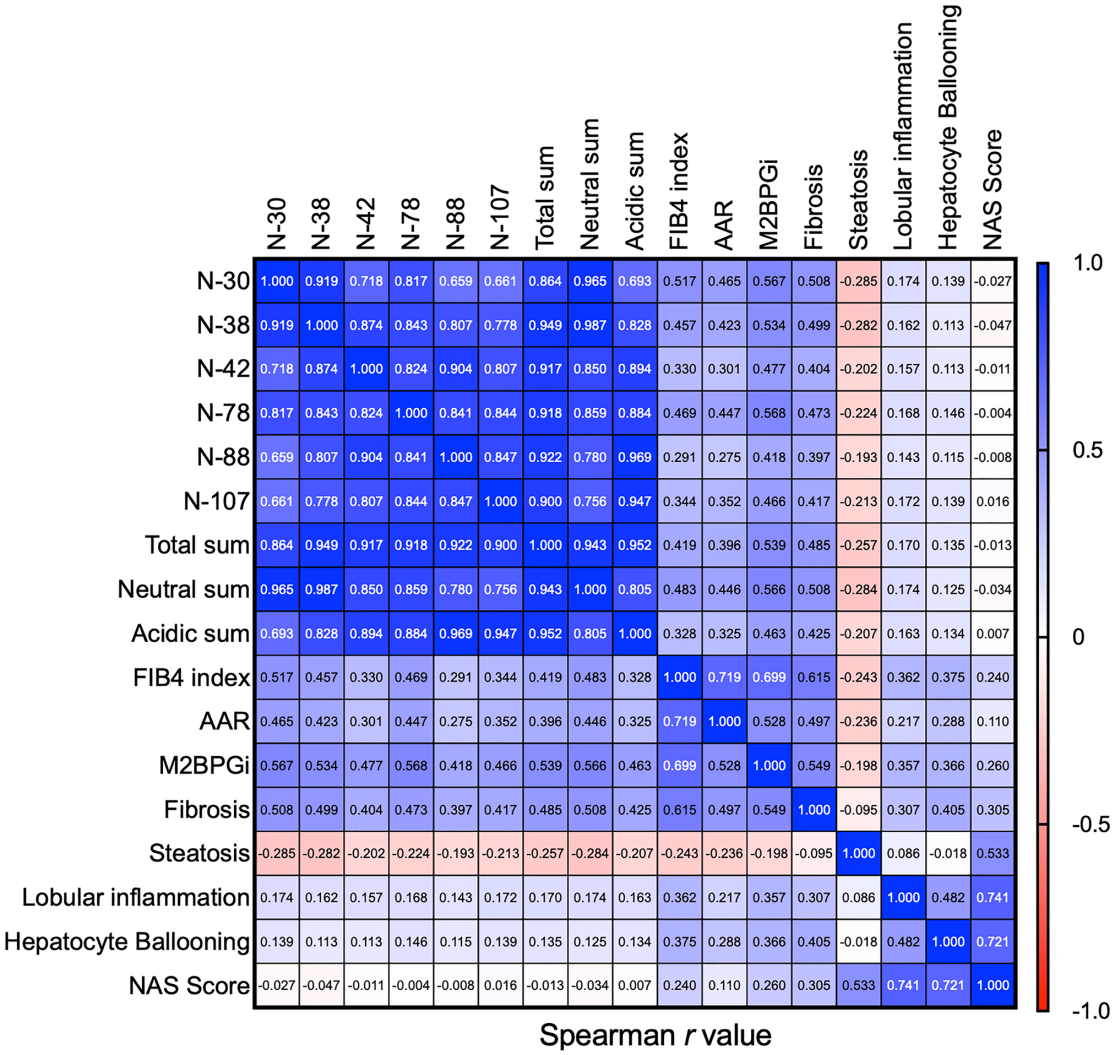
Correlation analysis of conventional fibrosis parameters and A2F bisect and its precursors *r* values calculated by Spearman’s correlation

The FIB4 index, the AAR, and M2BPGi levels are used as conventional parameters to evaluate the progression of liver fibrosis [24,27]. First, we examined the correlation between these conventional parameters of liver fibrosis and expression of A2F bisect and its precursors carrying bisecting GlcNAc and core fucose. As shown in Table 2, the AUC values of individual bisect-related *N*-glycans and the calculated sums were similar to or higher than those of conventional parameters of liver fibrosis, while expression of these glycans showed a weak correlation with conventional parameters (Fig. 2). Expression of these glycans also correlated with fibrosis stage, similar to conventional parameters.

**Fig. 3:**
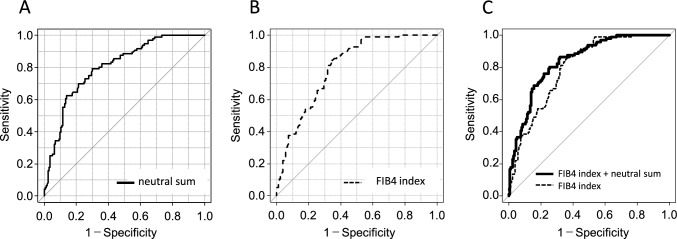
Diagnostic performance of the neutral sum, the FIB4 index, and a combination of these parameters, for advanced liver fibrosis. **A** ROC analysis of the neutral sum. **B** ROC analysis of the FIB4 index. **C** ROC analysis of the combined neutral sum and FIB4 index

Next, we examined the correlation between the expression of A2F bisect glycan and its precursors and pathological parameters such as the steatosis score, lobular inflammation score, hepatocyte ballooning score, and the summed value (NAS score). Expression of the selected glycan candidates correlated negatively with the steatosis score (similar to the FIB4 index, AAR, and M2BPGi). By contrast, expression levels of A2F bisect glycan, its precursors, and the calculated sum groups correlated weakly with the lobular inflammation score, and did not correlate significantly with the hepatocyte ballooning score or the NAS (Fig. 2). Therefore, we conducted multivariate regression analysis using variables independently associated with advanced fibrosis in univariate analysis and revealed that the FIB4 index (odds ratio (OR), 1.705; 95% confidence interval (CI), 1.291–2.252; *P* < 0.001) and the neutral sum (OR, 1.013; 95% CI, 1.007–1.019; *P* < 0.001) (Table 3) were significantly and independently associated with advanced fibrosis. The diagnostic performance of these combined variables was 0.840, which is better than either alone (0.804 and 0.792, respectively; Fig. 3).

**Table 3:** The factors associated with advanced fibrosis in patients with biopsy proven MASLD

	F0/1/2 (*n* = 173)	F3/4 (*n* = 96)	Univariate analysis	Multivariate analysis	Odds ratio
BMI (kg/m^2^)	29.6 ± 5.0	29.5 ± 3.9	0.912		
Albumin (g/dL)	4.3 ± 0.3	4.0 ± 0.3	< 0.001	0.997	
T-bil (mg/dL)	0.8 ± 0.3	0.9 ± 0.4	0.137		
AST (IU/L)	61 ± 30	60 ± 21	0.272		
LDL-C (mg/dL)	116 ± 28	104 ± 24	0.018	0.631	
AAR	0.80 ± 0.26	1.08 ± 0.25	< 0.001	0.968	
FIB4 index	1.80 ± 1.04	3.47 ± 1.49	< 0.001	< 0.001	1.705 (1.291–2.252)
M2BPGi (COI)	0.99 ± 0.54	1.97 ± 1.15	< 0.001	0.687	
A2F bisect	28.5 ± 18.2	55.7 ± 31.3	< 0.001	0.659	
Total sum	149.3 ± 72.7	279.5 ± 120.2	< 0.001	0.643	
Neutral sum	65.8 ± 34.9	129.5 ± 54.4	< 0.001	< 0.001	1.013 (1.007–1.019)

### Identification of carrier proteins bearing A2F bisect glycan and its precursors

Fibrosis biomarkers based on changes in protein-specific glycosyl expression may be more specific than markers in whole serum. Therefore, we attempted to identify glycan carrier proteins by focusing on A2F bisect glycan and its precursors. Initially, serum was fractionated into an eluted fraction and a flow-through fraction using Protein G Sepharose, followed by *N*-glycomic analysis of each fraction as previously described [28]. The *N*-glycome profiles of the eluted and flow-through fractions are shown in Supplementary Figure S2. In the eluted fraction, glycans derived from IgG (such as G0F (N-23), G1F (N-28), and G2F (N-36)) were enriched. By contrast, and unexpectedly, A2F bisect glycan (N-107), which is the final product in the biosynthetic pathway, was more abundant in the eluted fraction than in the flow-through fraction. Moreover, the eluted fraction contained precursors of N-107 carrying bisecting GlcNAc and core fucose (N-30, 38, 42, 78, and 88). Next, we tried to identify the carrier protein present in the eluted fraction of pooled serum from patients in group F3/4. Proteins in the eluted fraction were separated by SDS-PAGE, resulting in visualization of 21 major protein bands after Coomassie brilliant blue staining (Supplementary Figure S3). The protein from each band was extracted and subjected to *N*-glycan analysis; the results showed that 15 of the 21 protein bands contained *N*-linked glycoproteins. Furthermore, A2F bisect glycan was detected in only three protein bands (No. 9, 10, and 14), with approximately 75% being present in protein band No. 10 (Supplementary Figure S3). Band No. 10 also contained all the precursor glycans carrying bisecting GlcNAc and core fucose. We identified three types of protein by PMF and MS/MS analysis. The major proteins in band No. 9 were immunoglobulin heavy constant mu (IgM) and complement C3. Band No. 10 and 14 contained immunoglobulin heavy constant alpha 1 and 2 (IgA1 and 2) and complement C3, respectively (Supplementary Table S4). The *N*-glycan profiles of IgM and IgA were broadly consistent with those reported by other groups ^[[29–31]]^.

### Construction of simple ELISA system based on detection of bisect glycans on IgA for diagnosis of liver fibrosis.

**Fig. 4:**
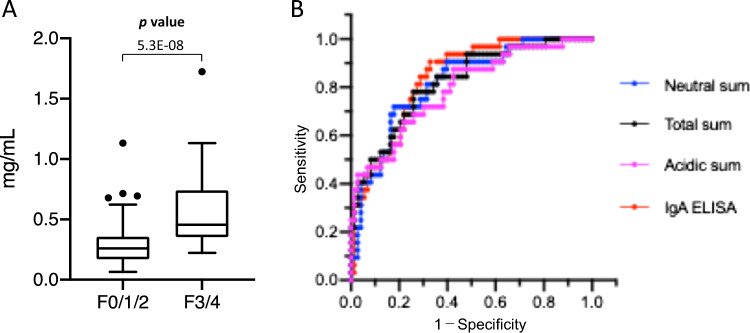
Diagnostic performance of the ELISA system based on an anti-human IgA antibody and PHA-E lectin for advanced liver fibrosis. **A** ELISA data for each fibrosis groups. **B** ROC analysis of the neutral sum (Blue), the total sum (Black), the acidic sum (Magenta), and the ELISA (Red)

Based on the results of A2F bisect glycan carrier protein identification, we constructed a sandwich ELISA system using an anti-human IgA antibody and PHA-E lectin, and the values of IgA bearing bisect glycans measured (bisect-IgA values). PHA-E lectin recognizes the bisecting GlcNAc structure, although the specific terminal sialic acids weaken the interaction [32]. ELISA was performed using serum samples from groups F0/1/2 (*n* = 73) and F3/4 (*n* = 32), and its diagnostic utility for advanced liver fibrosis was tested. The bisect-IgA values increased significantly in group F3/4 and correlated with the fibrosis stage (Fig. 4A and Supplementary Figure S5). Moreover, the bisect-IgA values showed a correlation with the total sum (*r* = 0.684), whereas they correlated weakly with lobular inflammation (*r* = 0.397) and the hepatocyte ballooning score (*r* = 0.255), which are pathological inflammatory parameters as shown in Supplementary Figure S5. The correlation between bisect-IgA values and the FIB4 index was also not strong (*r* = 0.546). ROC analysis revealed that the AUC of the established ELISA system was 0.838, higher than those of the total sum (AUC = 0.819), the neutral sum (AUC = 0.817), and the acidic sum (AUC = 0.793; Fig. 4B and Supplementary Table S5), but the difference was not statistically significant. We also developed another sandwich ELISA system using PHA-E lectin and an anti-human kappa light chain antibody that detects immunoglobulins. In this ELISA system, the AUC value was 0.801, which is slightly lower than that of the ELISA based on the anti-IgA antibody (Supplementary Figure S4 and Table S6).

## Discussion

MASLD is thought to affect 30% of the global population, and is considered an important type of liver disease in the post-viral era[33]; however, a lack of non-invasive, rapid, and low-cost methods means that diagnosis of advanced liver fibrosis is difficult. The FIB4 index is developed as a non-invasive scoring system based on routine tests to predict liver fibrosis in patients co-infected with HIV/HCV [34]. A previous study reported the utility of combining the FIB4 index with magnetic resonance elastography (MRE) [35]; however, few facilities offer MRE, and it is a costly and time-consuming test. Several approaches involving the detection of lectins bound to glycans on proteins have been investigated as innovative biomarkers of disease, and for clinical testing. In the context of liver disease, Wisteria floribunda agglutinin-positive M2BP (M2BPGi) appears to be useful for evaluating liver fibrosis in patients with viral hepatitis, autoimmune hepatitis, and MASLD [3,27,36–38].

Previously, we developed a comparative glycomic analysis method based on lactone ring-opening isotope labeling to identify α2,3-linked sialoglycans [21]. Alterations in α2,3-linked sialoglycans present in serum during progression of liver fibrosis were detected quantitatively by linkage-specific aminolysis; however, this analytical method is not suitable for α2,6-linked sialylated and neutral *N*-glycans. In the present study, we performed comprehensive and quantitative analyses of *N*-glycans using aminolysis-SALSA. We found that the expression of many *N*-glycans differed among the patient groups. Interestingly, we found that levels of A2F bisect *N*-glycan and its precursors increased significantly during liver fibrosis. In addition, we confirmed that the level of A2F bisect *N*-glycan, the total sum, and the neutral sum in healthy individuals without fatty liver were approximately equal to those in the F0 and F1 groups (Supplementary Figure S7). ROC analysis of A2F bisect-related glycans revealed that diagnostic performance was strongly associated with the levels of bisecting GlcNAc and core fucose structures. Moreover, the total amount of categorized *N*-glycans (Total sum: N-30, N-38, N-42, N-78, N-88, and N-107) and neutral *N*-glycans (Neutral sum: N-30, N-38, and N-42) was a better diagnostic indicator of liver fibrosis than conventional parameters (i.e., the FIB4 index, AAR, and M2BPGi).

The FIB4 index and M2BPGi reflect not only liver fibrosis but also other factors such as inflammation and liver injury [39–41]. When comparing these conventional markers with our glycan parameters, we found that the FIB4 index and M2BPGi also correlated with pathological inflammatory parameters such as lobular inflammation and the hepatocyte ballooning score. By contrast, we found that the expression levels of A2F bisect and its precursors carrying both bisecting GlcNAc and core fucose residues show similar tendencies during the progression of liver fibrosis, and are rarely associated with inflammation.

The cut-off value of the FIB4 index is strongly affected by age [42]. The FIB3 index subtracts the effects of age but needs more validation before use in routine clinical practice [43]. When we examined the diagnostic performance of the FIB4 index and the neutral sum for advanced liver fibrosis according to age, we found that the FIB4 index performed less well (AUC = 0.689) in those older than 60 years. By contrast, the diagnostic performance of the neutral sum was not affected by age (AUC = 0.791 at < 60 years; AUC = 0.786 at ≥60 years) (Supplementary Table S7). These results indicate that the neutral sum may be an age-independent marker of liver fibrosis. Moreover, A2F bisect-related glycans were not associated with inflammatory parameters, which improved the diagnostic performance for advanced liver fibrosis when the neutral sum was combined with the FIB4 index (AUC = 0.840).

Additionally, it was reported that the FIB4 index is less accurate at predicting liver fibrosis in patients with diabetes [44]. In this study, 153 patients (56.9%) had diabetes. The accuracy of the FIB4 index for predicting advanced liver fibrosis was relatively low in patients with diabetes (AUC = 0.771) compared with the entire cohort (AUC = 0.792) (Supplementary Figure S8). By contrast, the diagnostic accuracy of the neutral sum remained relatively high in patients with diabetes (AUC = 0.843) compared with the entire cohort (AUC = 0.804). Therefore, diabetes may affect the predictive accuracy of the FIB4 index, but not of the neutral sum, for advanced liver fibrosis. However, further analysis is needed to validate these findings.

Moreover, we further evaluated the diagnostic accuracy of A2F bisect glycan and its precursor glycans for the detection of F2 and F4 fibrosis. For F2 fibrosis, the diagnostic performance of A2F bisect glycan and its precursor glycans was comparable with those of the FIB4 index, M2BPGi, and AAR (Supplementary Table S8). For F4 fibrosis, while the sensitivity of A2F bisect glycan and its precursor glycans was higher than those of the FIB4 index, M2BPGi, and AAR, its AUC was slightly lower than those of certain other markers (Supplementary Table S9). However, the limited number of F4 cases in this analysis underscores the need for further studies with larger cohorts. Additionally, A2F bisect glycan and its precursor glycans demonstrated weak correlations with other markers and did not reflect inflammation (Fig. 2). This suggests that combining A2F bisect glycan and its precursor glycans with other markers may enhance diagnostic accuracy. We believe this warrants further investigation.

Previously, we developed the focused protein glycomics (FPG) procedure, which allows analysis of the glycan profiles of gel-separated serum proteins by MALDI-TOF MS, and identified unique glycoisoforms of vitamin D-binding protein and haptoglobin in STAM model mice with hepatocarcinogenesis [45]. In the present study, we attempted to use this method to identify the proteins that carry A2F bisect *N*-glycan, and identified IGHM, IGHA1 and 2, and complement C3. The *N-*glycan profiles on IGHM and IGHA proteins were broadly consistent with those reported previously [29–31]. Furthermore, IGHA1 and 2 carried high levels of A2F bisect glycan and its precursors, and the levels correlated strongly with liver fibrosis.

*N*-acetylglucosaminyltransferase III (MGAT3) is a glycosyltransferase that transfers GlcNAc to the core Man residue of *N*-glycans via a *β*1,4-linkage to form a bisecting structure. The Human Protein Atlas (https://www.proteinatlas.org) shows that MGAT3 activity is relatively high in the brain and kidneys. Activity of MGAT3 in the normal liver is nearly undetectable; however, its expression increases during hepatocarcinogenesis [46–48]. Additionally, MGAT3 activity in B cells, which produce IgA after differentiation into plasma cells, increases during liver fibrosis/cirrhosis [49,50]. Ochoa-Rios et al. reported that the levels of fucosylated and bisecting *N*-glycans are increased in human livers and model mice with non-alcoholic steatohepatitis [51]. Therefore, the progression of liver fibrosis in MASH may significantly affect the expression levels of bisecting *N*-glycan with core fucose on IgA.

We identified specific bisect *N*-glycans biosynthesized by MGAT3, and some of the carrier proteins, associated with the progression of liver fibrosis in patients with MASLD. About 75% of the detected A2F bisect glycan in whole serum was carried on IgA proteins. Many of glycans on IgA carried either bisecting GlcNAc or core fucose. The glycan alteration on IgA by MGAT3 is of great interest during progression of fibrosis in patients with MASH. The elucidation of mechanisms underlying progression of liver fibrosis associated with IgA for bisect *N*-glycans can lead to develop novel therapeutic approaches. McPherson et al. reported that secretion of serum IgA correlates positively with the fibrosis stage [52]. A recent study by Kotsiliti et al. reported that intestinal B cells induce metabolic activity in T cells, accompanied by increased secretion of IgA, in patients with MASH [53]. Focusing on the alteration of specific bisect glycan on IgA, we constructed a sandwich ELISA that combines anti-human IgA with PHA-E lectins that recognize the neutral bisecting GlcNAc structure (bisect-IgA values). The bisect-IgA values showed a high correlation with the neutral sum calculated by MS analysis. The diagnostic utility of this ELISA system for advanced liver fibrosis was also comparable with that of calculated sums such as the neutral sum, total sum, and acidic sum. The bisect-IgA values showed higher diagnostic performance than that of IgA for liver fibrosis. Although this ELISA needs to be validated using a large number of specimens, the system would be very useful for mass screening to identify patients with advanced fibrosis.

In this study, to ensure internal validity, we performed multivariate analysis to adjust for potential confounding factors such as age, sex, and BMI. These analyses allowed us to isolate the independent effect of A2F bisect *N*-glycan and its precursors as a biomarker for advanced fibrosis, minimizing the influence of other variables. Regarding external validity, our cohort of 269 liver biopsy cases included a diverse population with differences in the degree of liver fibrosis, age, sex, and BMI, supporting the generalizability of the findings. However, further studies of different populations would be beneficial to confirm the broader applicability of these findings. In addition, this study has several limitations. First, the sample size was relatively small because all cases included in the analysis were diagnosed through liver biopsy. Second, we were unable to evaluate the predictive accuracy for progression to hepatocellular carcinoma or decompensated liver cirrhosis. Third, due to the retrospective design of the study, several clinically relevant parameters could not be obtained. We were also unable to examine the difference in diagnostic performance of the enhanced liver fibrosis score (ELF score), an existing biomarker that is widely used worldwide. To address these limitations, larger, prospective studies are warranted.

In conclusion, this multicenter study identified A2F bisect *N*-glycan and its precursors as novel and highly accurate biomarkers for advanced fibrosis in patients with MASLD. We found that the expression levels of bisect glycans correlated weakly or rarely with lobular inflammation or hepatocyte ballooning. Combined analysis based on the calculated neutral sum (N-30, N-38, and N-42) and the FIB4 index showed improved diagnostic performance. Moreover, IgA1 and 2 were identified as carrier proteins for A2F bisect *N*-glycan, and a simple sandwich ELISA system using an anti-human IgA antibody and PHA-E lectin was able to diagnose the progression of liver fibrosis. The value of diagnostic performance using both sandwich ELISA and the FIB4 index also showed higher than using only one of each. Unlike conventional fibrosis biomarkers, the novel glycomarker reflects liver fibrosis more accurately without being affected by inflammation. Taken together, the glycan alteration bearing bisect GlcNAc on IgA may have the potential to serve as a novel diagnostic tool for MASLD in routine clinical practice.

## Supplementary Information

Below is the link to the electronic supplementary material.

Supplementary file1 (DOCX 3000 KB)
