# Supplementary material for: Elevated A2F bisect N-glycans of serum IgA reflect progression of liver fibrosis in patients with MASLD
Source: J Gastroenterol. 2025 Jan 24;60(4):456–68. doi: 10.1007/s00535-024-02206-8 (PMC11922979; doi:10.1007/s00535-024-02206-8)
Supplement: Supplementary file 1 — Supplementary file1 (DOCX 3000 KB) [file 535_2024_2206_MOESM1_ESM.docx]

Supplementary information for

**Elevated A2F Bisect Glycan and Its Precursors Reflect Progression of Liver Fibrosis and Development of a Simplified Assay System in Patients with MASLD**

**Table of contents**

Supplementary materials and methods...................................................................................2

Figure S1................................................................................................................................7

Figure S2................................................................................................................................8 Figure S3................................................................................................................................9

Figure S4..............................................................................................................................10

Figure S5..............................................................................................................................11

Figure S6..............................................................................................................................12

Figure S7..............................................................................................................................13

Figure S8..............................................................................................................................14

Table S1…............................................................................................................................15

Table S2…............................................................................................................................16

Table S3…............................................................................................................................17

Table S4…............................................................................................................................18

Table S5…............................................................................................................................19

Table S6…............................................................................................................................20

Table S7…............................................................................................................................21

Table S8…............................................................................................................................22

Table S9…............................................................................................................................23

**Supplementary materials and methods**

**Reagents**

The chemical 2-iodoacetamide (IAA) was purchased from FUJI FILM Wako Pure Chemical Corporation (Osaka, Japan). Trypsin and Tris(2-carboxyethyl) phosphine hydrochloride (TCEP) were purchased from Sigma-Aldrich (St. Louis, MO, USA). PNGase F PRIME was obtained from N-Zyme Scientifics (Doylestown, PA, USA). MultiScreen Solvinert 0.45 μm low-binding hydrophilic polytetrafluoroethylene plates were purchased from Merck Millipore (Darmstadt, Germany). MassPrep HILIC μElution plates were obtained from Waters Corporation (Milford, MA, USA). BlotGlyco beads were purchased from Sumitomo Bakelite Company (Tokyo, Japan). SialoCapper-ID Kit was purchased from Shimadzu Corporation (Kyoto, Japan). Amino-oxy-functionalized tryptophanylarginine methyl ester (aoWR) was prepared as previously described.^(1)^ Other solvents and reagents were of the highest grade commercially available.

**Extraction of glycoproteins from human serum**

450 μL of ethanol was added to 50 μL of serum and incubated at -30°C for 3 h. Supernatants and precipitated proteins were separated by centrifugation at 14,000 g for 20 min at 4°C. Then, 500 μL of ethanol was added to the precipitated proteins, and the mixture was separated by centrifugation and dried for 30 min at 37°C. The precipitated pellets were resolved in 100 μL of distilled water and used for *N*-glycan analysis.

**Preparation of *N*-glycans by glycoblotting combined with aminolysis-SALSA**

The precipitated protein pellets were treated with 500 mM TCEP and 100 mM 2-iodoacetamide in ammonium bicarbonate buffer containing 0.1% triton X-100. After reductive alkylation, proteins were digested with 10 mg/mL trypsin at 37°C for 3 h. The mixture was heated at 90°C for 15 min to inactivate trypsin. Deglycosylation was performed by the addition of 170 U of PNGase F PRIME (N-Zyme Scientifics, Doylestown, PA) and incubated at 37°C for 16 h. *N*-glycans corresponding to 2.5 μL of serum were subjected to glycoblotting combined with aminolysis-SALSA method. The glycan sample containing the internal standard disialyloctasaccharide (A2GN1; 50 pmol) was transferred to the wells of a MultiScreen Solvinert filter plate containing BlotGlyco beads (5 mg). After glycans capturing on beads, the well was washed with 2 M guanidine hydrochloride, H_2_O, 1% triethylamine in methanol. Then, unreacted hydrazide groups on the beads were acetylated with 10% acetic anhydride in methanol (MeOH). The beads were then washed with 10 mM HCl, MeOH, and dimethyl sulfoxide (DMSO), followed by addition of SialoCapper-ID Kit Reagent A and B to wells containing beads. The plate was then shaken in a microplate mixer (TOMY, Tokyo, Japan). After excess amidation solution was removed, the beads were washed with MeOH, SialoCapper-ID Kit Reagent C, MeOH, and H_2_O. Finally, glycans were recovered from the beads and labeled with aoWR by an imine exchange reaction. Excess aoWR reagent was removed using a HILIC elution plate prior to matrix-assisted laser desorption/ionization time-of-flight mass spectrometry (MALDI-TOF-MS) analysis.

**MALDI-TOF MS analysis**

A purified glycan solution was mixed with a 2,5-dihydrobenzoic acid solution (10 mg/mL in 30% MeCN) and subjected to MALDI-TOF MS analysis as previously described. Briefly, all measurements were performed on an Ultraflex II TOF/TOF mass spectrometer equipped with a reflector and controlled by FlexControl 3.0 software (Bruker Daltonics, Bremen, Germany). All spectra were obtained in reflectron mode with an acceleration voltage of 25 kV, a reflector voltage of 26.3 kV, and a pulsed-ion extraction of 160 ns in positive-ion mode. Masses were annotated using FlexAnalysis 3.0 software. Absolute quantitation was performed by comparing the areas of the MS signals derived from each *N*-glycan with a known amount of internal standard (A2GN1).

**Fractionation of serum using Protein G Sepharose**

Briefly, 20 μL of serum was diluted with 180 μL of binding buffer (20 mM sodium phosphate, pH 7.0) and then added to 50 μL of Protein G Sepharose (Merck). After rotation at 4°C for 1 h, the flow-through fraction was collected by centrifugation at 800 g at 4°C. Protein G Sepharose was washed with 200 μL of binding buffer. The Protein G Sepharose-bound fraction was eluted with 90 μL of elution buffer (0.1 M glycine-HCl, pH 3.0) and neutralized immediately with 10 μL of neutralization buffer (1 M Tris-HCl, pH 9.0).

**SDS-PAGE and in-gel tryptic digestion for peptide mass fingerprinting (PMF) analysis**

PMF analysis was performed as previously reported. Briefly, the Protein G Sepharose-eluted fraction (equivalent to 4 μL of serum) was applied to a precast gel (e-PAGEL, 5–20% polyacrylamide gel, ATTO, Tokyo, Japan) and separated by electrophoresis (300 V, 20 mA). The gel was stained with SimplyBlue SafeStain (Thermo Fisher Scientific). The stained protein bands were then excised and subjected to in-gel tryptic digestion for 16 h at 37°C. The peptides in the gels were extracted, hydrated in 200 μL of 5% formic acid, and purified on C18 ZipTip columns (Millipore, Bedford, MA, USA). The amino acid sequences were analyzed by MALDI-TOF MS and compared with the theoretical peptide masses in the MASCOT search engine (Matrix Science, Boston, MA, USA) to identify the protein species. The stained protein bands were also subject to in-gel PNGase F digestion for glycoblotting analysis of *N*-glycans.^(2)^

**ELISA for the detection of immunoglobulins bearing bisect *N-*glycans.**

Immunoglobulins carrying bisect *N*-glycans were quantified in a sandwich ELISA using a human IgA standard. The ELISA plates, Avidin Plate (Blocking-less type, Sumitomo Bakelite, Japan), were coated with PHA-E4-biotin (5 μg/mL; J-CHEMICAL, Japan) in 10% SuperBlock Blocking Buffer (Thermo Fisher Scientific) in PBS (pH 7.2) at 25°C for 30 min followed by washing three times with PBS, pH 7.2/0.05% Tween 20. 100 μL of serum samples (diluted 1:16000 in PBS, pH 7.2/0.05% Tween 20 or standard IgA (0–350 ng/mL in 1:16000 in PBS, pH 7.2/0.05% Tween 20) were added to the plate for 1 h at 25°C. The wells were washed three times with PBS, pH 7.2/0.05% Tween 20, and then incubated with 100 μL of anti-human Kappa light chain-HRP (Thermo Fisher Scientific) for 1 h at 25°C. For color development, TMB was added for 30 min at 25°C. After terminating the reaction with sulfuric acid, absorbance at 450 nm (OD450) was measured in a microplate reader. For correction, the OD630 value (reference absorbance at 630 nm) was subtracted from the OD450. The amount of the immunoglobulins carrying neutral bisect *N*-glycans were calculated from a calibration curve generated with human IgA.

**Supplementary references**

1. Uematsu R, Furukawa J, Nakagawa H, Shinohara Y, Deguchi K, Monde K, Nishimura S. High throughput quantitative glycomics and glycoform-focused proteomics of murine dermis and epidermis. Mol Cell Proteomics 2005;4:1977-1989.

2. Küster B, Wheeler SF, Hunter AP, Dwek RA, Harvey DJ. Sequencing of N-linked oligosaccharides directly from protein gels: in-gel deglycosylation followed by matrix-assisted laser desorption/ionization mass spectrometry and normal-phase high-performance liquid chromatography. Anal Biochem 1997;250:82-101.

**Figure S1**

Figure S1. The expression level of total sum, neutral sum, and acidic sum in each fibrosis groups. The results were expressed as the means ± S.D. *, 0.01<p<0.05; **, p<0.01

**Figure S2**

Figure S2. MALDI-TOF MS spectra of *N*-glycans in whole serum and Protein G fractionation samples.

a) Whole serum, b) flow-through fraction, c) eluted (Immunoglobulin) fraction equivalent to serum 2.5 μL. Red characters indicate that A2F bisect and precursors with bisecting GlcNAc and core fucose.

**Figure S3**

Figure S3. Separation of proteins in Protein G eluted fraction by SDS-PAGE and *N*-linked glycan analysis of protein bands. A. CBB staining of polyacrylamide gels. B. MALDI-TOF MS spectra of *N*-glycan in a) Protein band No.9, b) No.10, and c) No. 14. Red characters indicate that A2F bisect and its precursors with bisecting GlcNAc and core fucose.

**Figure S4**

Figure S4. Diagnostic performance of the ELISA system based on PHA-E lectin and an anti-human kappa-light chain antibody for advanced liver fibrosis. (A) ELISA data for each fibrosis groups. (B) ROC analysis of the neutral sum (Blue), the total sum (Black), the acidic sum (Magenta), and the ELISA (Red).

**Figure S5**

Figure S5. Spearman’s R correlation of conventional fibrosis parameters, IgA ELISA, and A2F bisect and its precursors. *r* values calculated by Spearman’s correlation analysis.

**Figure S6**

Figure S6. The diagnostic performance of IgA and constructed ELISA system using anti-IgA antibody and PHA-E in advanced liver fibrosis. (A) ROC analysis of IgA. (B) ROC analysis of ELISA system using anti-IgA antibody and PHA-E lectin. ROC analysis was performed in groups F0/1/2 (n=45) and F3/4 (n=23).

**Figure S7**

Figure S7. The expression level of A2F bisect, neutral sum, acidic sum, and total sum in Ctrl (n=8), F0 (n=9), F1 (n=8), F2 (n=4), F3 (n=11), and F4 (n=4).

**Figure S8**

Figure S8. The diagnostic performance of neutral sum and FIB4 index in patients with diabetes. (A) ROC analysis of neutral sum. (B) ROC analysis of FIB4 index. ROC analysis was performed in groups F0/1/2 (n=89) and F3/4 (n=64).

Table S1. The expression levels of *N*-glycans in several fibrosis classification groups

Table S2. List of N-glycan expression levels sorted by decreasing order of p-value in comparison between F0/1/2 and F3/4 groups.

Table S3. Diagnostic performance of A2F bisect precursors for discrimination of advanced liver fibrosis.

Table S4. List of peptide mass fingerprinting analysis of protein bands.

Table S5. Diagnostic performance of calculated sums and the anti-IgA antibody immobilized ELISA system in the context of advanced liver fibrosis

Table S6. Diagnostic performance of calculated sums and the PHA-E immobilized ELISA system in the context of advanced liver fibrosis

Table S7. Diagnostic performance of neutral sum and FIB4 index for discrimination of advanced liver fibrosis by age

Table S8. Diagnostic performance of A2F bisect, its precursors, and conventional markers for discrimination F2 liver fibrosis

Table S9. Diagnostic performance of A2F bisect, its precursors, and conventional markers for discrimination F4 liver fibrosis
